# A Hybrid Biofuel and Triboelectric Nanogenerator for Bioenergy Harvesting

**DOI:** 10.1007/s40820-020-0376-8

**Published:** 2020-02-14

**Authors:** Hu Li, Xiao Zhang, Luming Zhao, Dongjie Jiang, Lingling Xu, Zhuo Liu, Yuxiang Wu, Kuan Hu, Ming-Rong Zhang, Jiangxue Wang, Yubo Fan, Zhou Li

**Affiliations:** 1 Beijing Advanced Innovation Centre for Biomedical Engineering, Key Laboratory for Biomechanics and Mechanobiology of Chinese Education Ministry, School of Biological Science and Medical Engineering, Beihang University, Beijing, 100083 People’s Republic of China; 2 CAS Center for Excellence in Nanoscience, Beijing Key Laboratory of Micro-Nano Energy and Sensor, Beijing Institute of Nanoenergy and Nanosystems, Chinese Academy of Sciences, Beijing, 100083 People’s Republic of China; 3 School of Nanoscience and Technology, University of Chinese Academy of Sciences, Beijing, 100049 People’s Republic of China; 4 School of Physical Education, Jianghan University, Wuhan, 430056 People’s Republic of China; 5 Center on Nanoenergy Research, School of Physical Science and Technology, Guangxi University, Nanning, 530004 People’s Republic of China; 6 Department of Radiopharmaceuticals Development, National Institute of Radiological Sciences, National Institutes for Quantum and Radiological Science and Technology, Chiba, 263-8555 Japan

**Keywords:** Self-powered, Triboelectric nanogenerator, Glucose fuel cell, Hybrid energy harvester, Bioenergy

## Abstract

Various types of energy exist everywhere around us, and these energies can be harvested from multiple sources to power micro-/nanoelectronic system and even personal electronic products. In this work, we proposed a hybrid energy-harvesting system (HEHS) for potential in vivo applications. The HEHS consisted of a triboelectric nanogenerator and a glucose fuel cell for simultaneously harvesting biomechanical energy and biochemical energy in simulated body fluid. These two energy-harvesting units can work individually as a single power source or work simultaneously as an integrated system. This design strengthened the flexibility of harvesting multiple energies and enhanced corresponding electric output. Compared with any individual device, the integrated HEHS outputs a superimposed current and has a faster charging rate. Using the harvested energy, HEHS can power a calculator or a green light-emitting diode pattern. Considering the widely existed biomechanical energy and glucose molecules in the body, the developed HEHS can be a promising candidate for building in vivo self-powered healthcare monitoring system.

**Electronic supplementary material:**

The online version of this article (10.1007/s40820-020-0376-8) contains supplementary material, which is available to authorized users.

## Highlights

- A triboelectric nanogenerator (TENG) and a glucose fuel cell (GFC) were separately designed to harvest biomechanical energy from body motion and biochemical energy from glucose molecules.
- A hybrid energy-harvesting system (HEHS) which consisted of TENG and GFC was developed successfully, and it can simultaneously harvest biomechanical energy and biochemical energy.

**Electronic supplementary material:**

The online version of this article (10.1007/s40820-020-0376-8) contains supplementary material, which is available to authorized users.

## Graphical abstract

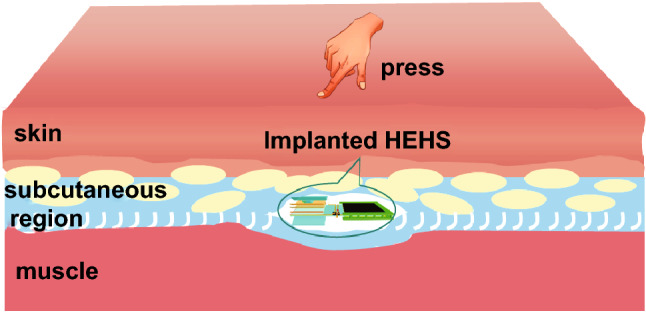

## Introduction

With the fast development of portable and implantable electronic devices, various new remarkable techniques of energy supplies experienced rapid growth. Relevant research studies involve energy generation, energy harvesting, and energy storage for micro-/nanoelectronic systems, which can be used to realize some specific functions [1–7]. Besides energy from nature and surroundings (e.g., wind energy and solar energy) [8–10], there are also many biomechanical and biochemical energy from body can be harvested (e.g., respiration, heart beating, and glucose oxidation) [11–17]. If these energies can be efficiently collected and stored, it will be possible to meet the energy requirements of many low-power electronic products, or even personal electronics [18, 19].

Triboelectric technology has been proved to be an effective means to harvest these ubiquitous energies and convert them into electricity, which can be used in health surveillance [19–23], cell/nerve stimulation [24–26], or even power a commercial cardiac pacemaker [27]. These achievements bring new insights for scientific researchers and doctors to diagnose and treat related diseases. When a triboelectric device was implanted in body, it collected energy from respiration [28], heart beating [27], blood flow [29], and so on. Whereas the biochemical energy (e.g., glucose) in body fluid around the device slipped away, if this energy can be simultaneously harvested, the converted electric energy will be augmented, and this step will likely be sufficient for powering microdevices in clinic, for instance, implantable wireless glucose sensor for diabetic patient [30], temperature monitor after surgery [31], and pressure sensor for arterial blockage [32]. Considering glucose in tissue fluid or blood, it is a feasible conception to use these biomolecules for glucose fuel cell (GFC) and generating electric energy by redox reaction [33].

In this study, we designed a hybrid energy-harvesting system (HEHS) by integrating a triboelectric nanogenerator (TENG) and GFC to simultaneously harvest the biomechanical energy and biochemical energy. The HEHS was integrated on a flexible polyethylene terephthalate (PET) substrate. TENG and GFC were connected in parallel, and their electric outputs were superimposed successfully, which proved its feasibility to harvest the mechanical energy and biochemical energy simultaneously. Compared with any single unit, the HEHS has a faster charging rate to a commercial capacitor, which proved its higher efficiency as a hybrid system to harvest energy. Then the harvested energy can power a commercial calculator and a green light-emitting dioxide (LED) pattern. This study provided a feasible method to harvest energy from multiple sources simultaneously, and it has a great potential as a power source to drive micro-/nanodevices to achieve some specific functions.

## Experimental Section

### Fabrication of TENG

Aluminum (Al) foil was fixed on a PET substrate by silver paste and acted as one friction layer. The Al foil was polished using sandpaper to create microstructures [34]. Kapton film with copper back electrode was selected as another friction layer. Precut polydimethylsiloxane (PDMS) (thickness, 2 mm) was used as spacer to make the friction layers keep a gap [4, 16]. One piece of titanium (Ti) foil was used as the backbone to ensure the fast recovery of friction layers after contact [29]. To protect TENG from water infiltration, it was encapsulated with polytetrafluoroethylene (PTFE) film and PDMS in sequence.

### Fabrication of Glucose Fuel Cell (GFC)

Bacterial cellulose (BC) membranes were purchased from Hainan Yida Food Co. Ltd., which were used as the matrix scaffold of multiwalled carbon nanotubes (MWCNTs). These BC membranes were pretreated in sodium hydroxide (NaOH) solution (0.1 mol L^−1^) at 90 °C for 1 h and then rinsed repeatedly with deionized (DI) water until neutral.

The dispersion of MWCNTs was prepared by dispersing MWCNTs (0.4 g) and sodium dodecylbenzene sulfonate (SDBS) (4 g) in DI water (400 mL) with ultrasonication (100 W, 60 Hz) for 2 hours [35–39]. The dispersion solution was centrifuged at 6000 rpm for 10 min. MWCNTs were inserted into BC membrane (BC/MWCNTs) by infiltrating the as-prepared supernatant to improve the conductivity of BC membranes. H_2_PtCl_6_·6H_2_O (1 g) was dissolved in DI water to prepare chloroplatinic acid solution. The samples should be protected from light. Palladium chloride was dissolved in 20 mM HCl solution (25 mL) at 60 °C for 1 h. BC/MWCNTs was soaked in the mixed solution of chloroplatinic acid and chloropalladium acid (volume ratio, 1:1) for 1 h and then put it in sodium borohydride solution (0.1 M) at 90 °C with stirring for 1 h to obtain BC/MWCNTs/Pt–Pd film; it was used as the anode film. BC film with MWCNTs on both sides served as cathode film.

Gold film with scheduled area was sputtered on polyethylene terephthalate (PET) substrate. The BC membrane clings to the gold film. A polylactic acid (PLA) fixture fabricated by 3D printing was used to fix the electrode film and supporting substrate. PBS solution was prepared by mixing sodium hydrogen phosphate (Na_2_HPO_4_·12H_2_O) and potassium phosphate monobasic (KH_2_PO_4_) in DI water. Glucose solution (1 g L^−1^) was added in PBS solution and used as the electrolyte of the cathode and anode electrode.

### Integration of GFC and TENG

GFC and TENG were integrated on a transparent and flexible PET substrate. Au films as cathode and anode for GFC were sputtered on both sides of PET. Al foil of TENG and Au film of GFC were isolated by a blank region to prevent them from conduction. A rectifier and a unilateral diode were connected to TENG and GFC, respectively. Then the rectified TENG and GFC were connected in parallel. The TENG was encapsulated with PTFE and PDMS to ensure that the HEHS can work normally in watery environment.

### Material Characterization and Electrical Measurement

To confirm the BC/MWCNTs/Pt–Pd has the ability to oxidize glucose, its electrochemical characteristic was tested by electrochemical workstation. BC/MWCNTs/Pt–Pd film was tied on a glassy carbon electrode as working electrode. Platinum electrode and calomel electrode (SCE) were used as counter electrode and reference electrode, respectively [40]. The materials’ properties were characterized by scanning electron microscope (SEM, HITACHI, SU8020) and X-ray diffraction (XRD, PANalytical, X’Pert^3^ Powder). The electrical outputs of TENG and GFC were measured by an electrometer (Keithley, 6517B) and a digital oscilloscope (Teledyne LeCroy, HDO6104). A commercial capacitor (capacity, 10 μF) was used to store energy of TENG, GFC, and HEHS, respectively.

## Results and Discussion

### Conception of Using HEHS for Multiple Energy Harvesting

Human body contains many types of energy, such as biomechanical energy, biochemical energy, and thermal energy. If these energies were effectively collected, it will be beneficial to provide electric energy for self-powered portable electronics. Because the thermal energy is difficult to be harvested due to the limitation of temperature difference in human body, the biomechanical energy and biochemical energy became the preferred candidates for energy conversion.

**Fig. 1:**
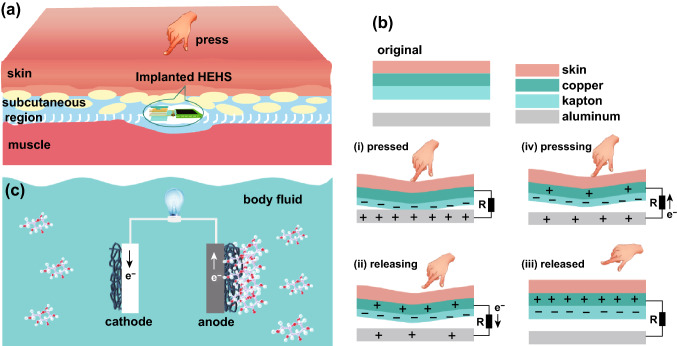
**a** Conception graph of an implanted HEHS harvesting biomechanical energy and biochemical energy in body. **b** Biomechanical energy-harvesting mechanism by TENG under the finger press. **c** Biochemical energy-harvesting mechanism by GFC from glucose molecules in body fluid

The biomechanical energy can be from external or internal body motions, for instance, finger pressing and hand flapping. The biochemical energy can be from the glucose molecules in body fluid. If a proposed HEHS was implanted in a suitable position, it will be feasible for the HEHS to simultaneously harvest biomechanical energy and biochemical energy from the body motion and surrounding body fluid (Fig. 1a). As shown in Fig. 1a, a HEHS was implanted in the subcutaneous region, and it was surrounded by body fluid. When a finger pressed on the skin, the local pressure brings friction materials (Kapton and aluminum) into contact [Fig. 1b(i)], equal amount of opposite charges distributed on their contact surfaces due to coupling of triboelectrification and electrostatic induction. When the finger gradually moves away from the skin, free electrons will migrate from copper to aluminum to balance the potential difference until the space recovered to the initial state [Fig. 1b(ii) and (iii)]. When the finger approaches the skin again, free electrons migrate from aluminum to copper until the Kapton fully contact with aluminum [Fig. 1b(iv) and (i)]. The repetitively press and release lead to periodic electric current output in external circuit. And the biomechanical energy was converted into electric energy by the TENG.

Meanwhile, the body fluid containing glucose molecules penetrates into active materials and participates in the redox reaction around the anode electrode of GFC (Fig. 1c). The lost electrons migrated from anode to cathode and were captured by dissolved oxygen in body fluid. This process converted the biochemical energy in glucose into electric energy. In the following part, we showed the HEHS design and demonstrated the feasibility of using HEHS to harvest biomechanical energy and biochemical energy simultaneously in simulated body fluid. The harvested energy was used to power a calculator and a green LED pattern.

### Harvesting Mechanical Energy by TENG

**Fig. 2:**
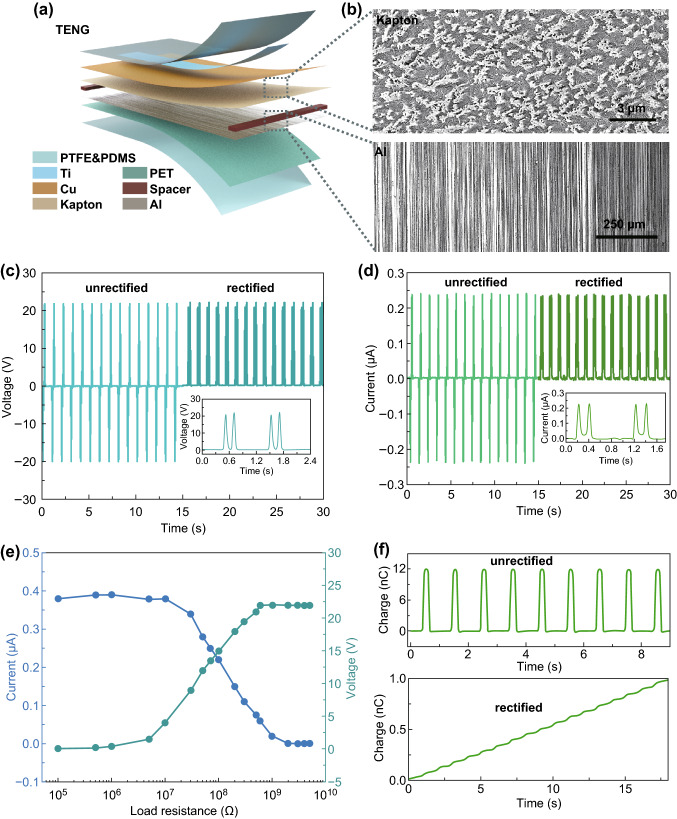
**a** Structure diagram of TENG. **b** Surface micromorphology of Kapton film and Al foil. **c** Working mechanism of TENG with a contact-separation mode. **d** Output voltage and **e** output current of TENG before and after rectification. **f** Transferred charge of TENG before (upper) and after (lower) rectification

The electric performance test of TENG was carried out in phosphate-buffered solution (PBS). The as-fabricated TENG has a vertical contact-separation mode. Kapton film and aluminum (Al) foil acted as friction layers. Thin copper layer was deposited on Kapton film as back electrode. The whole TENG device was packaged by polytetrafluoroethylene and polydimethylsiloxane (PTFE & PDMS) (Fig. 2a). The surface of Kapton film was treated by inductively coupled plasma-reactive ion etching (ICP) to form micropillars. The surface of Al foil was polished by sandpaper to form parallel microchannels. The microstructures on friction layers contribute to increasing the electric output of TENG (Fig. 2b).

As shown in Fig. 2c, the TENG can output an open-circuit voltage of about 22 V under the stimulus of a linear motor, and after rectification, the voltage value kept stable. The short-circuit current was about 0.24 μA, and after rectification, the current value has no decay (Fig. 2d). The effective output power of the TENG was measured by recording the voltage and current values with different load resistances ranging from 0.1 MΩ to 5 GΩ (Fig. 2e). The current decreased with increasing load resistance because of the Ohmic loss, while the voltage showed an increasing trend. A maximum power density of about 3.3 mW cm^−2^ was obtained with a load resistance of about 70 MΩ (Fig. S1). The transferred charge was about 12 nC without rectification in each cycle (upper, Fig. 2f). After rectification, the transferred charge was about 1 μC within 18 s (lower, Fig. 2f). These results indicated that the as-fabricated TENG can efficiently harvest biomechanical energy and convert it into electric energy.

### Preparation and Micromorphology of GFC

**Fig. 3:**
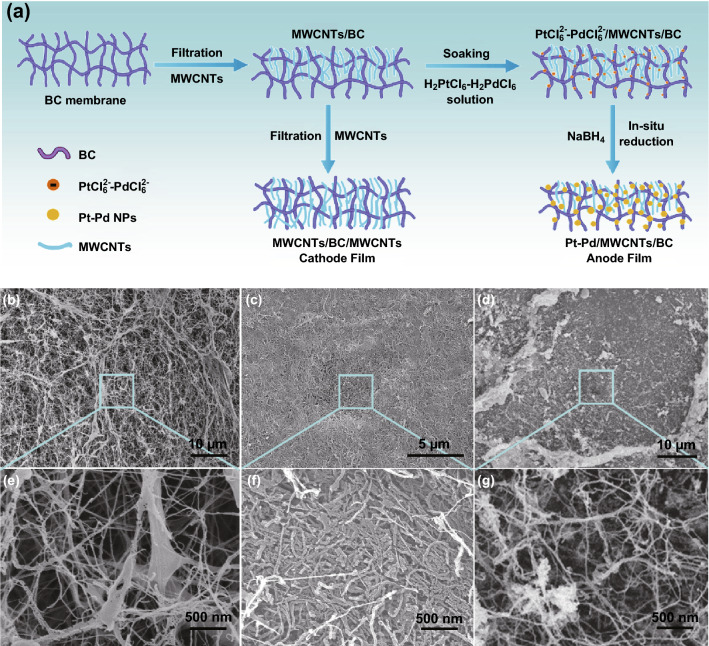
**a** Preparation process of cathode and anode films of GFC. **b, e** Surface micromorphology of original BC membrane. **c, f** Surface morphology of MWCNTs filtrated into BC membrane. **d, g** Micromorphology of Pt–Pd nanoparticles (NPs) on the surface of MWCNTs/BC film

To harvest the biochemical energy, redox reaction-based GFC was fabricated to convert the biochemical energy from glucose into electric energy. Bacterial cellulose (BC) membrane was selected as the supporting matrix scaffold due to its good biocompatibility and porous structure, which is in favor of biological application and high specific area for redox reaction. Figure 3a shows two types of BC membranes with MWCNTs prepared as the cathode and anode films, respectively. Firstly, MWCNTs were filtrated into both sides of a BC membrane, and it was named MWCNTs/BC/MWCNTs and used as the cathode film of GFC. Secondly, after one side of BC membrane was filtrated with MWCNTs, it was soaked in H_2_PtCl_6_–H_2_PdCl_6_ solution and then reduced by NaBH_4_ to obtain the catalyst Pt–Pd nanoparticles (NPs), the product was named Pt–Pd/MWCNTs/BC and used as the anode film of GFC (Figs. S2 and S3).

Before MWCNTs filtration, the original BC membrane has a porous structure, the filamentary BC interweaved with each other (Fig. 3b), and their edges were clear (Fig. 3e). This porous structure provided enough space for MWCNTs filling. After the filtration, BC membrane was filled with MWCNTs (Fig. 3c). MWCNTs in BC connected with each other (Fig. 3f), which ensured its conduction of electrons when redox reaction occurred. With the in situ reduction of Pt–Pd, many particles with various sizes appeared on the surface of MWCNTs/BC membrane, which indicated that the Pt–Pd catalyst was loaded successfully (Fig. 3d, g). XRD peaks of Pt–Pd of the as-prepared sample also verified its existence in MWCNTs/BC membrane (Fig. S4), which ensured the realization of redox reaction in the following experiment.

### Harvesting Biochemical Energy by GFC

**Fig. 4:**
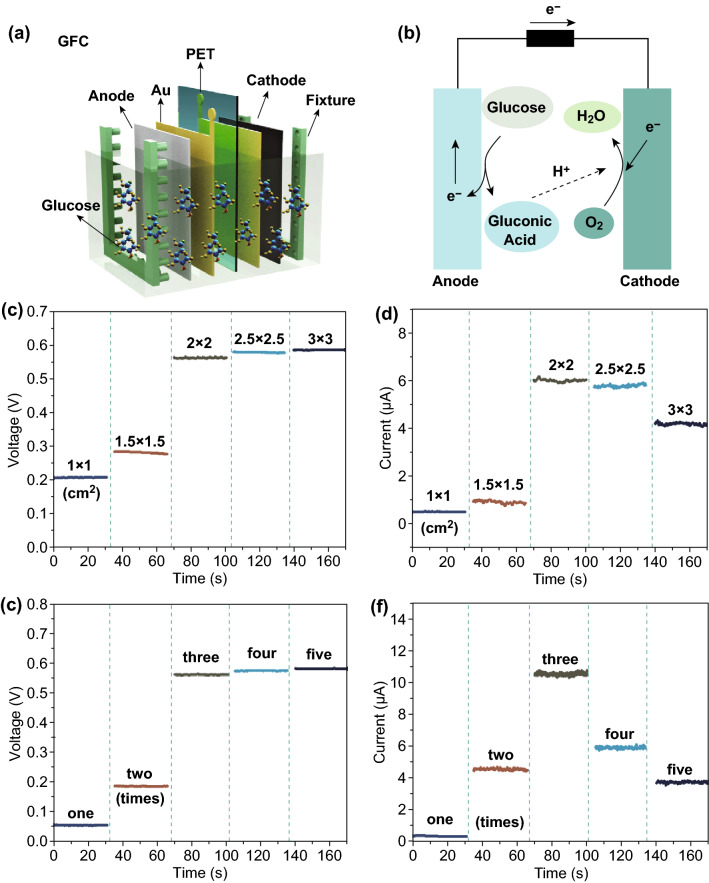
**a** Structure diagram of GFC in glucose solution. **b** Redox reaction of glucose at anode and cathode. **c, d** Variation of output voltage and current with different sizes. **e–f** Variation of output voltage and current with different loading times of Pt–Pd catalyst

As shown in Fig. 4a, a GFC was assembled layer by layer on a flexible PET substrate to harvest the biochemical energy from glucose molecules. The components include anode, gold (Au) electrode, PET substrate, cathode, and fixture. When the GFC was immersed into glucose solution, glucose molecules will be oxidized to gluconic acid at anode. Electrons flowed from anode to the cathode through the external circuit and generated electric current. The dissolved oxygen around cathode accepted electrons and combined with hydrogen ions to form water molecules (Fig. 4b). The redox reaction equations are as follows [41, 42]:

$${\text{Anode:}}\, {\text{C}}_{6} {\text{H}}_{12} {\text{O}}_{6} + {\text{ H}}_{2} {\text{O }} \to {\text{ C}}_{6} {\text{H}}_{12} {\text{O}}_{7} + \, 2{\text{H}}^{ + } + \, 2{\text{e}}^{ - }$$

$${\text{Cathode:}}\, 1/2{\text{O}}_{2} + \, 2{\text{H}}^{ + } + \, 2{\text{e}}^{ - } \to {\text{ H}}_{2} {\text{O}}$$

$${\text{Overall:}}\, {\text{C}}_{6} {\text{H}}_{12} {\text{O}}_{6} + \, 1/2{\text{O}}_{2} \to {\text{ C}}_{6} {\text{H}}_{12} {\text{O}}_{7} .$$

The cyclic voltammetry (CV) test of Pt–Pd/MWCNTs/BC was performed in PBS/glucose solution to prove the redox potential of GFC (Fig. S5c). When the glucose is absent, the CV curve has typical peaks for the hydrogen adsorption/desorption between − 0.6 and − 0.2 V versus SCE reference electrode. After adding the glucose in PBS, CV curve shows increased faradaic currents due to glucose oxidation in three regions. In anodic positive scan, glucose oxidation occurred in two regions, including the hydrogen desorption region from -0.6 to -0.4 V and the double-layer region from − 0.4 to 0.2 V. The third region for glucose oxidation occurred in the cathodic negative scan, i.e., hydrogen adsorption region from − 0.6 to 0.1 V [43]. These results indicated the qualified biochemical energy-harvesting ability of the as-fabricated GFC.

To obtain the optimum energy-harvesting performance, the device size and loading time of catalyst for GFC were studied. As shown in Fig. 4c, d, the output voltages and currents of GFCs with five sizes were discussed, i.e., 1 × 1, 1.5 × 1.5, 2 × 2, 2.5 × 2.5, and 3 × 3 cm^2^ (Figs. S6 and S7). The output voltage first increased rapidly with device size, and then it gets close to stable state after 2 × 2 cm^2^. This variation trend can be ascribed to the constant theoretical redox potential difference for glucose and oxygen [43]. With the size increase, more glucose molecules participate in the reaction, and the output voltage increased with the size and gradually approach theoretical value and then became stable. Because the internal resistance of GFC itself also shared the voltage, so its output voltage was slightly lower than 1 V. As for the output current, it firstly increased to about 6 μA at 2 × 2 cm^2^ and then decreased with the size. This trend can be attributed to the resistance variation with size. With increase in size, the effective reaction area and transferred electrons also rose, which contributes to the increase in output current. Meanwhile, the increased size will also raise the bulk resistance of GFC, contact resistance between anode/cathode and Au electrode, which will decrease the current. Under the combined action, the current showed increasing trend at the early stage, then decreased at later stage under the combined action. According to these results, the size of 2 × 2 cm^2^ was preferred in fabricating GFC.

To endow the GFC with ability of harvesting biochemical energy from glucose, Pt–Pd catalyst was loaded on MWNCTs/BC for different times. As shown in Fig. 4e, the output voltage of GFC firstly increased to about 0.6 V at three times of loading, and then it gets close to stable state with loading times. The voltage variation trend was similar to that shown in Fig. 4c. As for the output current, it firstly increased to 10.5 μA at three times of loading, then decreased with loading times (Fig. 4f). This decreasing trend can be attributed to the excessive loading of catalyst. The excessive loading will result in geometrical hindrance and self-poisoning effect, and it can slow the effective redox reaction [33, 43, 44]. According to the results, three times of loading was preferred in fabricating GFC.

### Integrated HEHS for Multiple Energy Harvesting

**Fig. 5:**
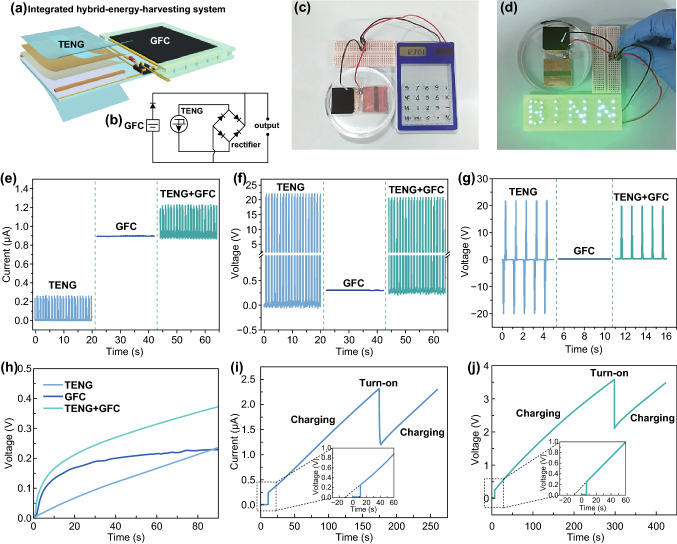
**a** Structure diagram and **b** circuit diagram of the integrated HEHS for multiple energy harvesting. Demonstration of powering **c** a calculator and **d** a green BINN pattern by the HEHS in PBS/glucose solution. **e** Output current of rectified TENG, GFC and their hybrid device. **f** Output voltage of rectified TENG, GFC and their hybrid device. **g** Output voltage of unrectified TENG, GFC, and their hybrid device (i.e., HEHS). **h** Charging curves by rectified TENG, GFC, and their hybrid device (i.e., HEHS). Charging/discharging curves of powering **i** a calculator and **j** a green BINN pattern

As demonstrated above, the as-fabricated TENG and GFC can work individually and harvest mechanical energy and biochemical energy. To prove the feasibility of using TENG and GFC to harvest multiple energies simultaneously, a hybrid energy-harvesting system (HEHS) consisted of TENG and GFC was developed and integrated on a flexible PET substrate (Fig. 5a). Considering that the voltage of TENG (22 V) was much higher than that of GFC (less than 1 V), and their output currents were similar, and therefore, the TENG and GFC were preferentially connected in parallel to enhance the combined output current. The TENG was rectified to generate unidirectional current. A unilateral diode was used to avoid the reverse charging from TENG to GFC (Figs. 5b and S8). As shown in Fig. 5e, the output currents of individual TENG and GFC were about 0.3 and 0.9 μA, respectively. After integrating the TENG and GFC, the current curve of TENG appeared on the current curve of GFC, and their currents were superimposed to about 1.2 μA successfully. The output voltages of individual TENG and GFC were 22 and 0.3 V (Fig. 5f), respectively. After integrating the TENG and GFC, the output voltage of TENG appeared on the voltage curve of GFC, and the peak voltage drop slightly to 21.7 V due to the parallel connection circuit (Fig. 5f). If the TENG was not rectified, only half number of peak voltages was retained after integrating the TENG and GFC. Similarly, the peak currents were also reduced by half due to reverse charging between TENG and GFC (Figs. 5g and S9).

When use the rectified TENG, GFC and their hybrid device (i.e., HEHS) to charge a capacitor, respectively, the HEHS has an obviously faster charging rate than TENG and GFC, it can obtain a higher voltage (0.37 V) than that (0. 24 V) of TENG and GFC within 90 s (Fig. 5h). To demonstrate the feasibility of using the HEHS to power portable electronics, a capacitor was charged to 2.3 (Fig. 5i) and 3.6 V (Fig. 5j), respectively, and it can provide energy for a calculator (Fig. 5c) and light up a green BINN pattern (Fig. 5d) immediately. Additionally, from the enlarged view in Fig. 5i and j, the charged voltage can quickly reach to about 0.3 V due to the existence of direct-current GFC, which can save about 20 s from the gray extension line.

## Conclusions

In summary, the fabrication parameters of TENG and GFC were studied in detail. Three times of Pt–Pd loading and 2 × 2 cm^2^ were selected as the preferential parameters for GFC fabrication. The developed TENG and GFC can effectively harvest biomechanical energy and biochemical energy, respectively. The HEHS can simultaneously harvest biomechanical energy and biochemical energy in simulated body fluid (i.e., PBC/glucose solution). Before integrating the HEHS, TENG and GFC should be rectified to protect the circuit from reverse charging and enhance the overall energy conversion ability. After integrating the HEHS in parallel, the output currents and voltages of TENG and GFC were superimposed successfully. When used the HEHS to convert the mechanical energy and biochemical energy into electric energy and stored in a capacitor, a portable calculator and a green LED pattern were powered successfully. Based on these results, this study provided a feasible method to harvest energy from multiple sources, and it is reasonable to think that the HEHS can be a promising candidate when implanted into the body to harvest biomechanical and biochemical energy simultaneously. The HEHS has a potential as a power source to drive low-power electronic devices to achieve some specific functions.

## Electronic supplementary material

Below is the link to the electronic supplementary material.

Supplementary material 1 (PDF 523 kb)
