# Correction to: A Hybrid Biofuel and Triboelectric Nanogenerator for Bioenergy Harvesting

**DOI:** 10.1007/s40820-020-00433-1

**Published:** 2020-04-16

**Authors:** Hu Li, Xiao Zhang, Luming Zhao, Dongjie Jiang, Lingling Xu, Zhuo Liu, Yuxiang Wu, Kuan Hu, Ming-Rong Zhang, Jiangxue Wang, Yubo Fan, Zhou Li

**Affiliations:** 1 Beijing Advanced Innovation Centre for Biomedical Engineering, Key Laboratory for Biomechanics and Mechanobiology of Chinese Education Ministry, School of Biological Science and Medical Engineering, Beihang University, Beijing, 100083 People’s Republic of China; 2 CAS Center for Excellence in Nanoscience, Beijing Key Laboratory of Micro-Nano Energy and Sensor, Beijing Institute of Nanoenergy and Nanosystems, Chinese Academy of Sciences, Beijing, 100083 People’s Republic of China; 3 School of Nanoscience and Technology, University of Chinese Academy of Sciences, Beijing, 100049 People’s Republic of China; 4 School of Physical Education, Jianghan University, Wuhan, 430056 People’s Republic of China; 5 Center on Nanoenergy Research, School of Physical Science and Technology, Guangxi University, Nanning, 530004 People’s Republic of China; 6 Department of Radiopharmaceuticals Development, National Institute of Radiological Sciences, National Institutes for Quantum and Radiological Science and Technology, Chiba, 263-8555 Japan

## Correction to: Nano-Micro Lett. (2020) 12:50 10.1007/s40820-020-0376-8

In the original publication, the authors' contribution is missing in the acknowledgment section. The correct acknowledgement is provided in this correction. Also, in Fig. 4, the second (c) after figure (d) should be read as (e). In Fig. 5 (i), the Y-axis label “Current (μA)” should be read as “Voltage”.

