# Supplementary material for: A Hybrid Biofuel and Triboelectric Nanogenerator for Bioenergy Harvesting
Source: Nanomicro Lett. 2020 Feb 14;12:50. doi: 10.1007/s40820-020-0376-8 (PMC7770853; doi:10.1007/s40820-020-0376-8)
Supplement: Supplementary file 1 — Supplementary material 1 (PDF 523 kb) [file 40820_2020_376_MOESM1_ESM.pdf]

Supporting Information for

A hybrid Biofuel and Triboelectric Nanogenerator for Bioenergy Harvesting

Hu Li[1, 2], Xiao Zhang[1, 2], Luming Zhao[2, 3], Dongjie Jiang[2, 3], Lingling Xu[2, 3], Zhuo Liu [1, 2], Yuxiang Wu[4], Kuan Hu[6], Ming-Rong Zhang[6], Jiangxue Wang[1, *], Yubo Fan[1, *], Zhou Li[2, 3, 5, *]

[1]Beijing Advanced Innovation Centre for Biomedical Engineering, Key Laboratory for Biomechanics and Mechanobiology of Chinese Education Ministry, School of Biological Science and Medical Engineering, Beihang University, Beijing 100083, People's Republic of China

[2]CAS Center for Excellence in Nanoscience, Beijing Key Laboratory of Micro-Nano Energy and Sensor, Beijing Institute of Nanoenergy and Nanosystems, Chinese Academy of Sciences, Beijing 100083, People's Republic of China

[3]School of Nanoscience and Technology, University of Chinese Academy of Sciences, Beijing 100049, People's Republic of China

[4]School of Physical Education, Jianghan University, Wuhan 430056, People's Republic of China

[5]Center on Nanoenergy Research, School of Physical Science and Technology, Guangxi University, Nanning 530004, People's Republic of China

[6]Department of Radiopharmaceuticals Development, National Institute of Radiological Sciences, National Institutes for Quantum and Radiological Science and Technology, Chiba 263-8555, Japan

*Corresponding authors. E-mail: yubofan@buaa.edu.cn (Yubo Fan); wangjiangxue@buaa.edu.cn (Jiangxue Wang); zli@binn.cas.cn (Zhou Li)

Supplementary Figures

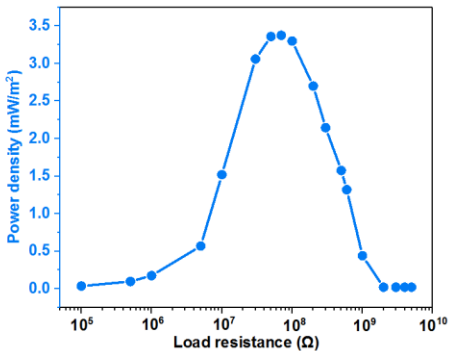

Fig. S1 The dependence of power density on the external load resistance

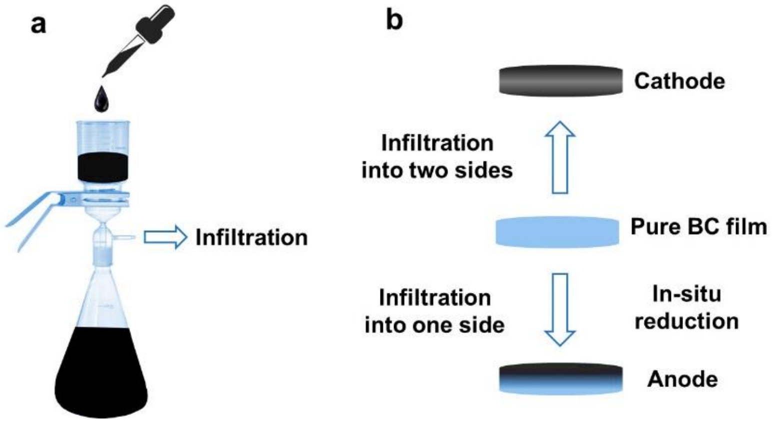

Fig. S2 Infiltration diagram of cathode and anode of GFC

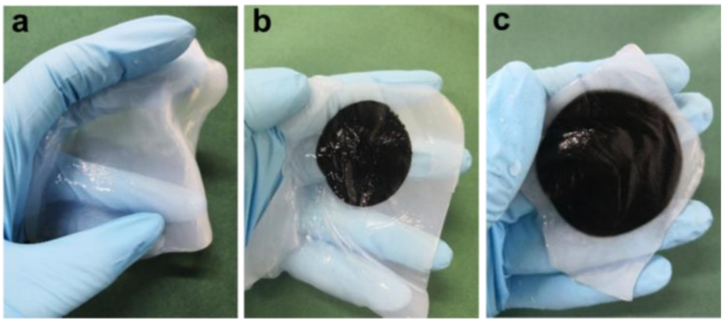

Fig. S3 Images of (**a**) pure BC film, (**b**) Pt-Pd/MWCNTs/BC film and (**c**) MWCNTs/BC/MWCNTs film

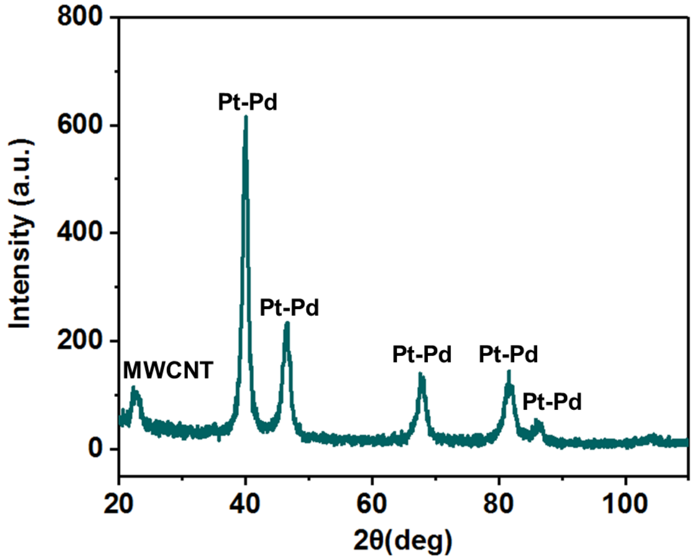

Fig. S4 XRD pattern of Pt-Pd/MWNCTs/BC

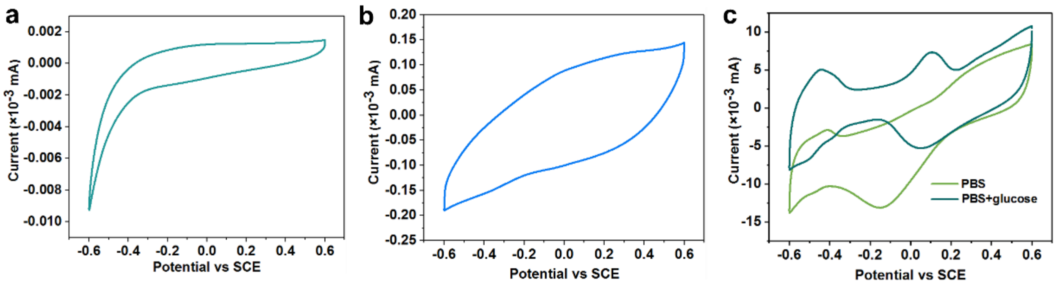

Fig. S5 CV test of (**a**) pure BC film and (**b**) MWCNTs/BC film in PBS solution at 10 mV s^{-1} with glucose. (**c**) CV test of Pt-Pd/MWNCTs/BC film in PBS solution at 10 mV s^{-1} in the absence (green curve) and the presence (cyan curve) of glucose

The CV curves of the pure BC film and MWCNTs/BC film showed capacitive characteristics without redox peaks under external applied voltage from -0.6 to +0.6 V (Fig. S5a, b). The CV curves of Pt-Pd/MWCNTs/BC film showed pseudocapacitive characteristics with redox peaks (Fig. S5c), which proved its ability of oxidizing glucose molecules.

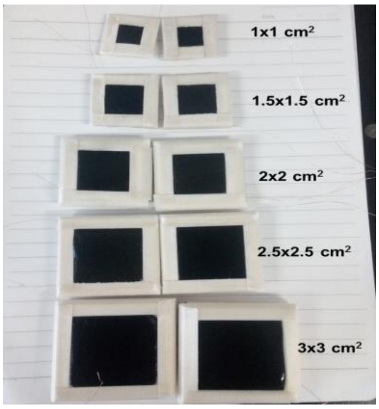

Fig. S6 Independent GFCs with different sizes from 1 × 1 to 3 × 3 cm^2 for performance test

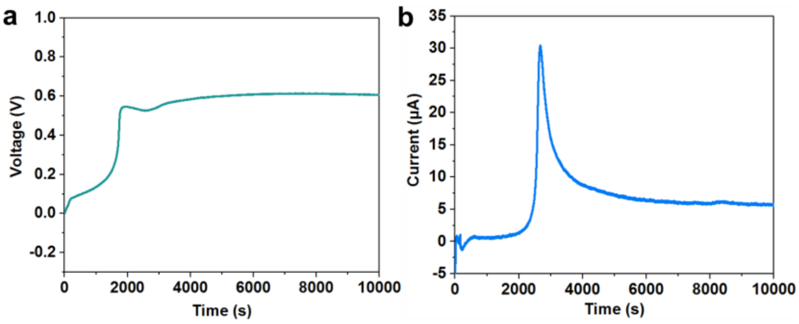

Fig. S7 (**a**) A typical output voltage curve and (**b**) a current curve of GFC with a size of 2 cm ×2 cm. The voltage value and current value were obtained at its stable stage after 5000 s (e.g., 0.6 V and 6 μA)

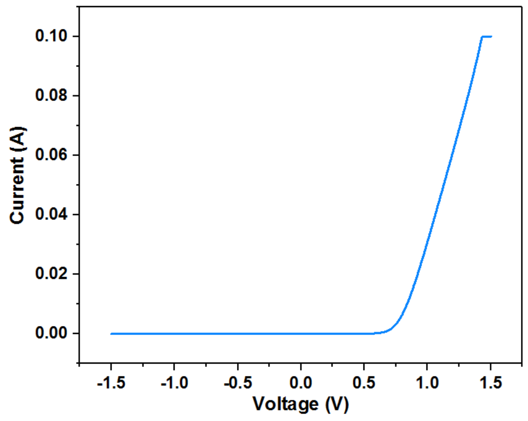

Fig. S8 Rectification character of the used unilateral diode. When the applied voltage was between -1.5 V to 0 V, the current was near to zero. When the applied voltage was between +0.5 V to + 1.5 V, the current rapidly increased to amperes. This asymmetric I-V curve indicated a good rectifying ability of the unilateral diode, which can effectively prevent the reverse charging

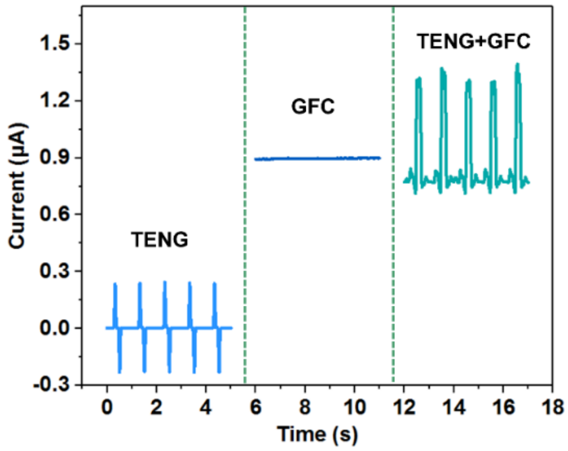

Fig. S9 Output current of unrectified TENG, GFC, and their hybrid device
